# Optic Radiation Tractography and Vision in Anterior Temporal Lobe Resection

**DOI:** 10.1002/ana.22619

**Published:** 2012-03-23

**Authors:** Gavin P Winston, Pankaj Daga, Jason Stretton, Marc Modat, Mark R Symms, Andrew W McEvoy, Sebastien Ourselin, John S Duncan

**Affiliations:** 1 1 Society Magnetic Resonance Imaging Unit, Department of Clinical and Experimental Epilepsy, University College London Institute of NeurologyLondon, United Kingdom; 2 2 College London Centre for Medical Image ComputingLondon, United Kingdom; 3 3 of Neurosurgery, National Hospital for Neurology and NeurosurgeryLondon, United Kingdom

## Abstract

**Objective:**

Anterior temporal lobe resection (ATLR) is an effective treatment for refractory temporal lobe epilepsy but may result in a contralateral superior visual field deficit (VFD) that precludes driving in the seizure-free patient. Diffusion tensor imaging (DTI) tractography can delineate the optic radiation preoperatively and stratify risk. It would be advantageous to incorporate display of tracts into interventional magnetic resonance imaging (MRI) to guide surgery.

**Methods:**

We studied 20 patients undergoing ATLR. Structural MRI scans, DTI, and visual fields were acquired before and 3 to 12 months following surgery. Tractography of the optic radiation was performed on preoperative images and propagated onto postoperative images. The anteroposterior extent of the damage to Meyer's loop was determined, and visual loss was quantified using Goldmann perimetry.

**Results:**

Twelve patients (60%) suffered a VFD (10–92% of upper quadrant; median, 39%). Image registration took <3 minutes and predicted that Meyer's loop was 4.4 to 18.7mm anterior to the resection margin in these patients, but 0.0 to 17.6mm behind the resection margin in the 8 patients without VFD. The extent of damage to Meyer's loop significantly correlated with the degree of VFD and explained 65% of the variance in this measure.

**Interpretation:**

The optic radiation can be accurately delineated by tractography and propagated onto postoperative images. The technique is fast enough to propagate accurate preoperative tractography onto intraoperative scans acquired during neurosurgery, with the potential to reduce the risk of VFD. ANN NEUROL 2012;

Up to 40% of patients with temporal lobe epilepsy (TLE) are refractory to medication,^1^ and anterior temporal lobe resection (ATLR) is an established and effective treatment.^2^ The benefits of surgery must be weighed against the possible adverse effects from damage to eloquent gray matter and white matter connections. The optic radiation conveys visual information from the lateral geniculate nucleus (LGN) to the primary visual cortex. Fibers representing the inferior visual field course directly posteriorly from the LGN, whereas those representing the superior field first pass anteriorly over the roof of the temporal horn of the lateral ventricle before turning backward (Meyer's loop).

Meyer's loop is vulnerable to damage during ATLR, with between 48%^3^ and 100%^4^ of patients experiencing a postoperative contralateral superior quadrantanopia that precludes driving in 4 to 50% of patients even if seizure free.^5^–^7^ Preservation of vision is key, as driving is among the most important goals for patients undergoing epilepsy surgery.^8^

The optic radiation shows high anatomical variability^9^ and cannot be delineated on conventional magnetic resonance imaging (MRI) sequences. Diffusion tensor imaging is an MRI technique that allows noninvasive in vivo delineation of tissue microstructure.^10^ By sensitizing the magnetic resonance signal to the degree of water diffusion, the predominant direction of diffusion can be determined in each brain voxel. Tractography algorithms are then used to trace white matter pathways by following the predominant direction of diffusion.

Tractography of the optic radiation can be used preoperatively to determine the distance between the temporal pole and Meyer's loop.^11^ This distance and the degree of resection are predictive of the extent of postoperative visual field deficit (VFD).^12^ As the optic radiation cannot be identified visually during surgery, the use of tractography data during image-guided surgery should reduce the risk of a VFD. However, preoperative tractography cannot be used, as brain shifts of up to 11mm occur following craniotomy.^13^

This study was designed to demonstrate the feasibility of a rapid novel image processing technique to propagate preoperative tractography data onto postoperative structural images by showing that the extent of damage to Meyer's loop as assessed by this technique is more highly predictive of visual field outcome following surgery than assessment using the combination of the degree of resection and location of Meyer's loop.

This method can next be applied to register preoperative tractography results to intraoperative structural MRI scans taken after craniotomy to make these data available in real time during image-guided surgery and reduce the risk of optic radiation damage.

## Patients and Methods

### Subjects

We studied 20 patients (age range, 17–56 years; median, 38 years; 11 male) with medically refractory TLE undergoing ATLR at the National Hospital for Neurology and Neurosurgery, London, United Kingdom. All patients had structural MRI scans performed at 3T,^14^ video electroencephalographic (EEG) telemetry, neuropsychology, neuropsychiatry, and if necessary intracranial EEG recordings prior to surgery. Structural MRI scans, diffusion tensor imaging (DTI), and visual fields were acquired before surgery and at a median of 4 months (range, 3–12 months) following surgery. Patients with pre-existing VFDs were excluded.

The study was approved by the National Hospital for Neurology and Neurosurgery and the Institute of Neurology Joint Research Ethics Committee, and informed written consent was obtained from all subjects. Patient demographics and clinical data are listed in the Table.

**Table:** Patient Demographics, Age of Epilepsy Onset, MRI Findings, Histological Diagnosis, ILAE Outcome at 1 Year (Where Available), and Postoperative VFD

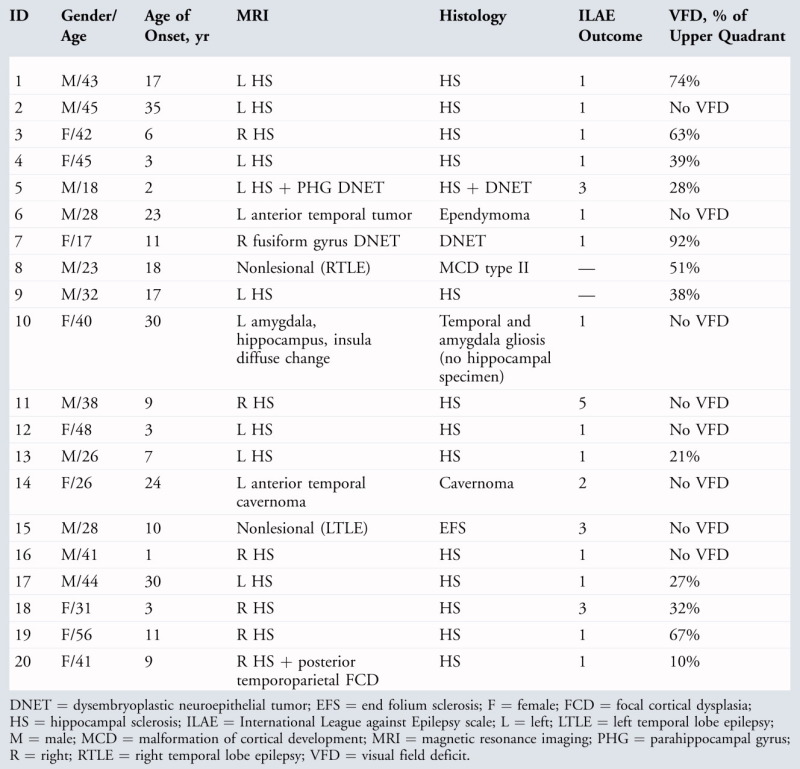

### Magnetic Resonance Data

MRI studies were performed on a 3T GE Excite II scanner (General Electric, Waukesha, Milwaukee, WI). Standard imaging gradients with a maximum strength of 40mT m^−1^ and slew rate 150T m^−1^ s^−1^ were used. All data were acquired using a body coil for transmission, and 8-channel phased array coil for reception. Standard clinical sequences were performed, and the coronal T1-weighted volumetric acquisition with 170 contiguous 1.1mm-thick slices (matrix, 256 × 256; in-plane resolution, 0.9375 × 0.9375mm) was used for the subsequent analysis.

### DTI Acquisition

DTI data were acquired using a cardiac-triggered single-shot spin-echo planar imaging sequence^15^ with echo time = 73 milliseconds. Sets of 60 contiguous 2.4mm-thick axial slices were obtained covering the whole brain, with diffusion sensitizing gradients applied in each of 52 noncollinear directions (*b* value of 1,200mm^2^ s^−1^ [δ = 21 milliseconds, Δ = 29 milliseconds, using full gradient strength of 40mT m^−1^]) along with 6 nondiffusion weighted scans. The gradient directions were calculated and ordered as described elsewhere.^16^ The field of view was 24cm, and the acquisition matrix size was 96 × 96, zero filled to 128 × 128 during reconstruction, giving a reconstructed voxel size of 1.875mm × 1.875mm × 2.4mm. The DTI acquisition time for a total of 3,480 image slices was approximately 25 minutes (depending on subject heart rate).

### DTI Preprocessing

The scans were transferred to a Linux-based Sun Ultra workstation in DICOM format and converted to a single multivolume Analyze 7.5 format file using locally written software. Eddy current correction of the DTI data was performed using the eddy correct tool in FSL (version 4.0.1).^17^

A multitensor model was fitted to the eddy corrected diffusion data using the Camino toolkit (version 2, release 767).^18^ Voxels in which a single tensor fitted the data poorly were identified using a spherical-harmonic voxel-classification algorithm.^19^ In such voxels, a 2-tensor model was fitted, with the principal diffusion directions of the 2 diffusion tensors providing estimates of the orientations of the crossing fibers. In all other voxels, a single tensor model was fitted.

### Tractography

Tractography was carried out using the Probabilistic Index of Connectivity algorithm^20^ as implemented in Camino extended to deal with multiple fibers.^21^, ^22^ Seed, way, and exclusion masks were defined as previously described.^12^

In brief, a 15-voxel seed region across the base of Meyer's loop was defined with a way point in the lateral wall of the occipital horn of the lateral ventricle and a midline exclusion mask. Tracking from the seed was performed using 50,000 Monte Carlo iterations, an angular threshold of 180°, and a fractional anisotropy threshold of 0.1, to ensure that the paths detected would not erroneously enter areas of cerebrospinal fluid, and yet had sufficient angular flexibility to allow tracking of Meyer's loop.

Finally, a coronal exclusion mask was used to remove artifactual connections to adjacent white matter tracts, such as the fronto-occipital fasciculus, anterior commissure, and uncinate fasciculus. An objective, iterative process was performed to determine the optimum location for this mask, whereby the exclusion mask was moved posteriorly until it began to coincide with Meyer's loop, identified by a visible thinning of the estimated trajectory of the optic radiation, typically associated with a reduction in tract volume >10%.^12^

A connectivity distribution was generated from each voxel in the seed region and combined into an overall connectivity map representing the maximum observed connection probability to each voxel within the brain from all the voxels within the seed region. For display purposes, the connectivity distributions were thresholded at 5%, representing a compromise between retaining anatomically valid tracts and removing obviously artifactual connections.

### Resection Size Estimates and Meyer's Loop Location

All resections were carried out by a single surgeon (A.W.M.), who employed a modified Spencer approach, localizing the lateral ventricles by proceeding from the middle cranial fossa floor up the collateral sulcus. Tractography data were not available during surgery. The anteroposterior distance from the temporal pole to the anterior margin of Meyer's loop (TP-ML distance) was determined using the preoperative non–diffusion-weighted image and the tractography results. The anteroposterior extent of resection was estimated by measuring the distance from the anterior tip of the middle cranial fossa to the posterior margin of resection on a postoperative sagittal image running through the lateral wall of temporal horn.^4^ These 2 measurements were expressed as a fraction of the anteroposterior distance from the temporal pole to the occipital pole (TP-OP) to account for differences in head size.^12^

### Propagation of Tractography and Measuring Damage

A novel 2-step image registration technique to perform accurate registration of preoperative to postoperative images was utilized.^23^ Automatic skull-stripping was performed on the postoperative images using the Brain Extraction Tool^24^ to ensure that the registration was driven only by brain tissue.

First a global image registration step was performed using a multiscale block-matching algorithm.^25^ This technique is robust to presence of outliers and leads to an accurate registration even in the presence of missing data, as in postoperative images. The affine transformation parameters to bring the images into global alignment were estimated and used to initialize the local registration step.

Next a local registration step was used to estimate the local, nonlinear geometric transformations. This was based on the free-form deformation algorithm,^26^, ^27^ with a transformation parameterized using uniform cubic B-splines. A bivariate normalized mutual information similarity measure was used, combining the structural MRI and fractional anisotropy images.^23^ This leads to a more accurate registration, as it uses information from both gray and white matter and exploits the shared information between diffusion and structural MRI images. The bending energy of the spline was added as a penalty term to ensure a smooth transformation.

**FIGURE 1:**
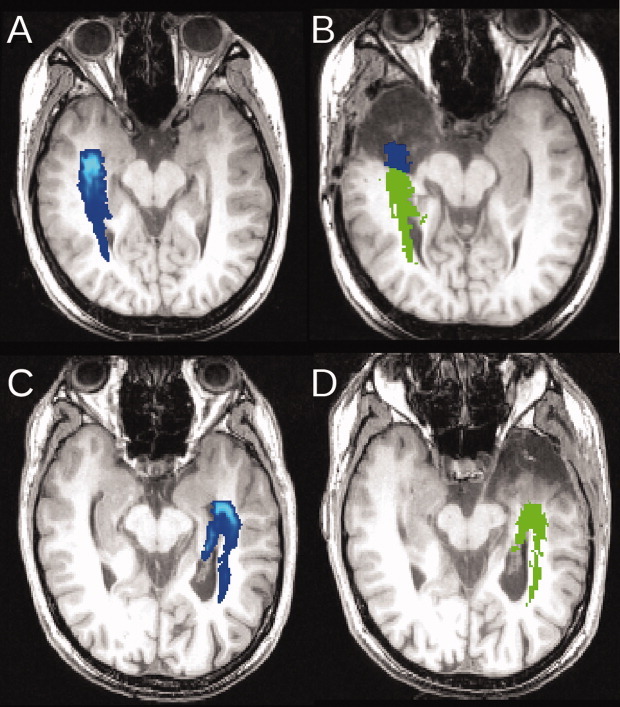
Preoperative structural T1-weighted image and optic radiation (A) and postoperative structural T1-weighted image with propagated preoperative tractography (B) show that part of the optic radiation was resected (blue) in patient 7, who developed a severe visual field deficit (VFD). Corresponding preoperative (C) and postoperative images (D) are shown in patient 12, who did not develop a VFD.

**FIGURE 2:**
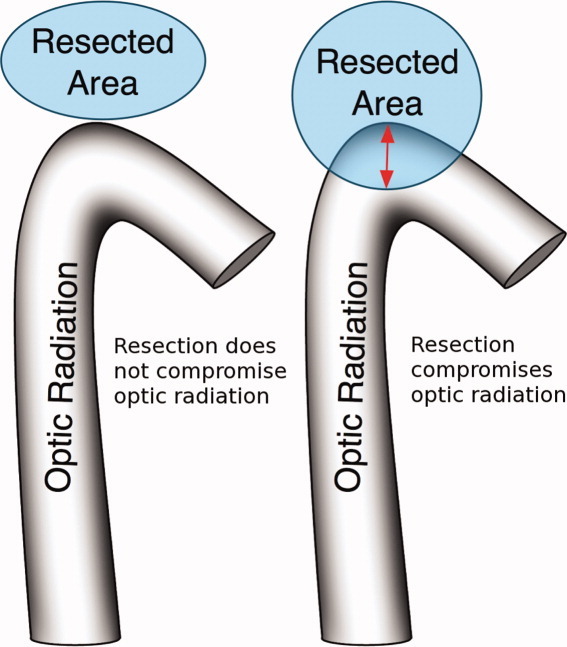
Measurement of the anteroposterior extent of resection of Meyer's loop.

The estimated transformation parameters were used to propagate the preoperative tractography representation of the optic radiation on the postoperative T1-weighted images (Fig 1). The amount by which Meyer's loop had been resected according to this registration was determined by measuring the anteroposterior distance between the most anterior portion of Meyer's loop and the resection margin on an axial slice (Fig 2).

### Visual Field Assessment

Postoperative visual fields were assessed in all patients using Goldmann perimetry, with 3 isopters (V4e, I4e, I2e) for every 15 degrees in each eye. The first 10 patients had visual fields assessed by confrontation prior to surgery (as the perimeter was not yet available), and the second 10 had preoperative Goldmann perimetry.

To determine the postoperative visual deficit, the postoperative visual fields were scanned, and the total area within the largest V4e isopter in each quadrant was determined by a locally written software application. Due to the high variability observed between Goldmann perimetry sessions^28^ and the lack of preoperative data in some patients, visual field loss was calculated using the areas of the upper quadrants (UQs) as follows: VFD = 1 − (area contralateral UQ [left eye] + area contralateral UQ [right eye])/(area ipsilateral UQ [left eye] + area ipsilateral UQ [right eye]).

Using the unaffected upper quadrant ipsilateral to the side of surgery as the reference for each patient allowed the use of postoperative data alone and eliminated intersession variability. No significant asymmetry in the upper quadrants was observed on preoperative Goldmann perimetry, and no deficits within the ipsilateral upper quadrants were observed in postoperative fields.

The number of patients who would not be permitted to drive due to the VFD was determined in accordance with UK Driver and Vehicle Licensing Agency regulations,^29^ with additional binocular Esterman perimetry if necessary.

### Statistical Analysis

Statistical analyses were performed using SPSS Statistics 17.0 (SPSS Inc, Chicago, IL). Two-sample *t* tests were used to compare the degree of resection and the TP-ML distances between patients not suffering a VFD and those with a postoperative VFD.

Two models were then used to model the relationship between the VFD and explanatory variables. The first model was a multiple regression analysis with VFD as the dependent variable and the TP-ML distance and resection size as the independent variables, both expressed as a fraction of the TP-OP distance. This model has previously been shown to be effective.^12^ Partial regression plots were inspected for outliers and heteroscedasticity, and only patients with a VFD were included, as the degree of VFD cannot fall below zero for smaller resections.

The second model was a regression analysis with predicted damage to Meyer's loop as the single independent variable, as this encompassed both the variability in the resection size and the location of Meyer's loop. If the image registration process is effective, this should explain an equal or greater amount of the variance in the observed VFD.

## Results

Twelve patients (60%) developed a postoperative VFD (10–92% of the upper quadrant; median, 39%). In these patients, visual criteria for driving were satisfied in 5 patients but were not met in 6 patients. The final patient declined Esterman perimetry, not wishing to drive, but on the basis of Goldmann perimetry would be very likely to meet the criteria. The resection size was 9.8 to 55.9% of the TP-OP distance (mean 31.2%). The largest resection (patient 8) was an outlier, with the next largest resection being 39.6%. Patient 8 had a nonlesional MRI, with right posterior temporal discharges on intracranial EEG, and thus underwent an ATLR, which was extended significantly posteriorly. The mean TP-ML distance was 32.1mm (range, 26.3–37.5mm). The size of resection was significantly greater in those developing a VFD than those without a VFD (36.1% vs 23.7%; 2-sample *t* test, *p* = 0.002). However, the location of Meyer's loop did not differ between those who did and did not develop VFD (mean TP-ML distance, 26.2% vs 25.7%, 32.6mm vs 31.7mm). These findings were not altered by exclusion of the outlier.

**FIGURE 3:**
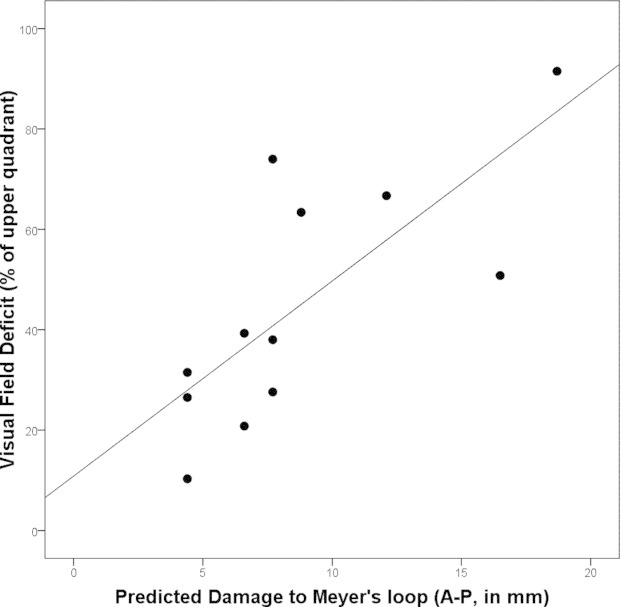
Correlation between measured visual field deficit and the predicted damage to Meyer's loop by image registration. A-P = anterior-posterior.

Propagation of preoperative tractography onto the postoperative images using the novel image registration technique showed that Meyer's loop lay 0.0mm to 17.6mm (mean, 4.0mm) behind the resection margin in the 8 patients without a postoperative VFD. In the 12 patients developing a VFD, the resection extended to between 4.4mm and 18.7mm (mean, 8.8mm) posterior to the anterior limit of Meyer's loop. There was a significant correlation between this anteroposterior distance and the degree of visual loss (Spearman correlation coefficient, 0.79; 1-tailed *p* = 0.001; Fig 3).

A multiple regression model with preoperative TP-ML distance and resection size (corrected for head size) as the explanatory variables for the VFD was strongly influenced by an outlier identified on partial regression plots (patient 8). Further review revealed that the resection margin was gradually sloping inferiorly toward the more posterior extent of the resection, with the optic radiation running in the roof. Therefore, the estimate of the anteroposterior extent of resection greatly overestimated the potential damage to Meyer's loop.

With this patient excluded, the model explained only a small part of the variance of the VFD (adjusted *R*^2^, 0.28; regression *p* = 0.11). In contrast, the simple regression model involving only the predicted damage to Meyer's loop via image registration explained much more of the variance in the VFD (adjusted *R*^2^, 0.65, regression *p* = 0.002). For each additional 1mm of damage to Meyer's loop, a further 5.0% of vision in the upper quadrant was lost. Furthermore, this model remained highly significant when the outlier was included, because the measurement of predicted postoperative damage to Meyer's loop looks only at the axial plane in which Meyer's loop lies and is not subject to the same error.

By implementation of the algorithm on graphical processing units,^30^ the mean registration time between pre- and postoperative images was 2 minutes, 55 seconds. This makes the technique suitable for use in a neurosurgical setting.

## Discussion

### Key Findings

The rationale of this study was to prove the possibility of registering preoperative tractographic representations of the optic radiation to postoperative scans in individual patients and to validate this technique by comparison with the VFD caused by surgery. Accurate registration explaining the majority of the variance in the measured postoperative VFD could be achieved within 3 minutes. This technique will next be applied intraoperatively using interventional MRI, so that the preoperative representation of the optic radiation is correctly displayed to the operating surgeon, taking account of the brain shift that occurs during surgery and facilitating the modification of the surgical approach to avoid damaging this critical pathway.

### Comparison with Previous Work

In a group of 21 patients undergoing ATLR, multiple linear regression analysis demonstrated that the preoperative TP-ML distance as assessed both by probabilistic tractography and by postoperative resection size was predictive of postoperative VFD on Goldmann perimetry.^12^ This model was therefore used as a comparator for the present study, and the new image registration technique was performed superiorly to this.

This previous study also suggested a greater risk of VFD with a lower TP-ML distance. In contrast, the present study shows no difference in the TP-ML distance between patients developing a VFD and those who do not. The size of resection was, however, significantly greater in patients with a postoperative VFD. This discrepancy may result from a more heterogeneous patient group, including a lower proportion of pure hippocampal sclerosis and the resulting increased variability in resection size (10–56%). There was little overlap in the anteroposterior extent of the resection between those not developing a VFD (13.2–38.5mm) and those developing a VFD (33.0–78.1mm).

In 48 patients undergoing ATLR, pre- and intraoperative imaging including both structural and diffusion scans showed that the postoperative VFD significantly correlated with damage to Meyer's loop as assessed by intraoperative tractography.^13^ However, there were significant limitations in this study. First, the tractography algorithm was a deterministic algorithm, and such algorithms underestimate Meyer's loop and are subject to error in regions of noise and crossing fibers. A direct comparison of deterministic and probabilistic algorithms in 18 subjects showed a TP-ML distance of 32 to 51mm (mean, 41mm) using a deterministic algorithm and 17 to 42mm (mean, 30mm) with a probabilistic algorithm.^31^ Anatomical dissection suggests a range of 22 to 37mm (mean, 27mm).^9^ Second, the visual outcome and damage to Meyer's loop were categorical and not true quantitative data. Finally, the intraoperative data were not available in real time to alter the surgical technique. Although preoperative tractography could be rigidly registered to the intraoperative images, this did not account for brain shift.

Preoperative deterministic tractography superimposed on the head-on surgical display has been used to guide entry into the temporal horn for selective amygdalohippocampectomy,^32^ but following this entry brain shift from cerebrospinal fluid leakage precludes further image guidance. This with gravity, cerebral edema, and the surgical procedure itself leads to an overall shift in the optic radiation of up to 11.1mm horizontally and 7.8mm vertically in either direction.^13^ Shift can only be accounted for using the suggested approach of acquiring and processing the tractography data prior to surgery and then using a comparison of pre- and intraoperative images to update the tractography results to align this with the intraoperative anatomy.

### Implications for Driving

Driving is the key activity that a VFD may preclude. In the UK “a field of at least 120° on the horizontal” with “no significant defect in the binocular field which encroaches within 20° of fixation above or below the horizontal meridian” is required,^29^ but regulations vary between countries. In 24 patients undergoing ATLR (20 with hippocampal sclerosis) from 1986 to 1995, 25% failed UK driving criteria,^6^ but in a subsequent larger study of 105 patients (91 with hippocampal sclerosis) at the same center covering 1998 to 2004, only 4% failed the same criteria.^7^ Another study found 50% of 14 patients failed to meet driving criteria following ATLR for hippocampal sclerosis, although in 2 patients this was due to prior vigabatrin usage.^5^

Assessment for driving in the United Kingdom typically uses automated binocular Esterman perimetry, which has been shown to be less sensitive for VFD and more lenient than the monocular Goldmann perimetry employed in the present study,^6^ and the European Union Eyesight Working Group has recommended against its use within Europe.^33^ Furthermore, all these studies concentrate on historical series in patients with predominantly hippocampal sclerosis. Improved imaging techniques and changes in surgical practice have enabled surgery in patients where it was not previously possible, including neocortical or nonlesional epilepsy.^34^ In the present series, only 12 patients had pure hippocampal sclerosis, with the remainder being dual pathology (n = 2), other pathology (n = 4) or nonlesional (n = 2). In such patients, more extensive resections may be proposed, with a corresponding greater risk of VFD, which needs to be assessed. In the patients studied, a total of 6 patients (30%) failed to meet UK criteria for driving.

### Strengths and Weaknesses

This study uses probabilistic tractography and a previously proven algorithm to ensure an accurate depiction of Meyer's loop of the optic radiation. Because neither rigid nor affine registrations account for the complex deformations seen during neurosurgery, a nonlinear registration technique is used. Such a technique must be both accurate and computationally rapid. High accuracy is achieved by combining the complementary information from anatomical and diffusion scans in the registration process. The average transfer time of a patient between scanner and operating table in the interventional MRI suite in the National Hospital for Neurology and Neurosurgery, London is 7 to 10 minutes. Implementation of the technique on graphical processing units reduced computation time to under 3 minutes, well within the required timescale, while retaining an accurate result.

This study has limitations. The degree of damage to Meyer's loop cannot be measured directly as a gold standard and has been inferred from preoperative imaging and a registration algorithm to postoperative images. It is not possible to perform tractography on all postoperative diffusion images as a comparator, as the tractography technique relies on a seed region across the base of Meyer's loop, a region that may have been resected during the surgery leading to a VFD. Comparisons of preoperative and postoperative tractography are also limited by variability within tractography resulting from minor changes in the seed location.^35^ The nature of the relationship between the damage to Meyer's loop and the degree of VFD is unknown, and the statistical analysis within this paper has assumed linearity as an approximation on the basis of the results obtained and previous studies. Measurement of a single anteroposterior distance is a relatively crude measure of damage to a complex structure such as the optic radiation; however, this is the quantity of most importance clinically for visual outcome.

The pre- and postoperative data were both of high quality and obtained on the same scanner. Intraoperative data will be acquired on a different scanner, which through limitations imposed both by design and by the time constraints of neurosurgery will yield images with lower spatial resolution and signal-to-noise ratio. We will use a high-field (1.5T) closed-bore Siemens Espree scanner, which provides superior image quality to conventional low-field “double-donut” design interventional magnetic resonance scanners. The signal-to-noise ratio is sufficient to facilitate the use of the proposed intensity-based image registration algorithm. Image registration is performed using an iterative coarse-to-fine approach in which the coarser levels can recover the larger deformations and ensure that the registration algorithm is more robust to noise by smoothing input images. The bivariate normalized mutual information similarity measure utilizes complementary and shared information generated by structural and diffusion MRI and so provides additional spatial constraints in the registration process. We have previously shown that this leads to better overall alignment.^23^, ^30^

As DTI data are acquired using an echo-planar imaging sequence, magnetic field inhomogeneities may cause geometric distortion at air–tissue interfaces such as the temporal pole, thereby introducing additional inaccuracies into image registration. The need for distortion correction and the application of the algorithm to intraoperative data must be evaluated, which is the subject of ongoing work.

### Implications of Results

Despite advances in surgical technique, VFDs remain common following temporal lobe epilepsy surgery. This study confirms the biological variability in the location of Meyer's loop and that increasing degrees of damage to this structure lead to greater VFD. Rather than just using preoperative tractography data to try to predict the risk of damage to vision, making these data available during surgery using accurate image registration will be a significant advance. Real-time display in a neuronavigation suite of the location of the optic radiation will be highly beneficial in avoiding surgical damage.

The validity of a suitable image registration technique has now been confirmed and can next be applied to intraoperatively acquired scans to guide surgery on an individual basis. The optic radiation will be superimposed on the intraoperative scans or the operating microscope display. Questions to be answered in this future work include the surgical margin of safety to add around the estimated location of the optic radiation and whether the provision of this additional information reduces the incidence or severity of VFD.
